# Soluble Ectodomain of Neuroligin 1 Decreases Synaptic Activity by Activating Metabotropic Glutamate Receptor 2

**DOI:** 10.3389/fnmol.2017.00116

**Published:** 2017-05-03

**Authors:** Michelle D. Gjørlund, Eva M. M. Carlsen, Andreas B. Kønig, Oksana Dmytrieva, Anders V. Petersen, Jacob Jacobsen, Vladimir Berezin, Jean-François Perrier, Sylwia Owczarek

**Affiliations:** ^1^Laboratory of Neural Plasticity, Department of Neuroscience and Pharmacology, University of CopenhagenCopenhagen, Denmark; ^2^Neuronal Signaling Laboratory, Department of Neuroscience and Pharmacology, University of CopenhagenCopenhagen, Denmark; ^3^Department of Biology, University of CopenhagenHelsingør, Denmark

**Keywords:** synaptic activity, proteolytic cleavage, cell adhesion molecule, hippocampus

## Abstract

Synaptic cell adhesion molecules represent important targets for neuronal activity-dependent proteolysis. Postsynaptic neuroligins (NLs) form trans-synaptic complexes with presynaptic neurexins (NXs). Both NXs and NLs are cleaved from the cell surface by metalloproteases in an activity-dependent manner, releasing a soluble extracellular fragment and membrane-tethered C-terminal fragment. The cleavage of NL1 depresses synaptic transmission, but the mechanism by which this occurs is unknown. Metabotropic glutamate receptor 2 (mGluR2) are located primarily at the periphery of presynaptic terminals, where they inhibit the formation of cyclic adenosine monophosphate (cAMP) and consequently suppress the release of glutamate and decrease synaptic transmission. In the present study, we found that the soluble ectodomain of NL1 binds to and activates mGluR2 in both neurons and heterologous cells, resulting in a decrease in cAMP formation. In a slice preparation from the hippocampus of mice, NL1 inhibited the release of glutamate from mossy fibers that project to CA3 pyramidal neurons. The presynaptic effect of NL1 was abolished in the presence of a selective antagonist for mGluR2. Thus, our data suggest that the soluble extracellular domain of NL1 functionally interacts with mGluR2 and thereby decreases synaptic strength.

## Introduction

The development and function of neuronal synapses depend on bidirectional interactions between pre- and postsynaptic components (Sanes and Lichtman, 1999). Presynaptic neurexins (NXs) and postsynaptic neuroligins (NLs) form an intercellular junction that is important for normal development, maintenance and function of synapses (reviewed by Südhof, 2008). Through interactions with their binding partners, the NX/NL complex triggers signal transduction at both excitatory and inhibitory synapses (reviewed by Bang and Owczarek, 2013). Deletions of human NX genes might be involved in the pathology of schizophrenia whereas NL gene mutations are indicated in schizophrenia (Zhang Z. et al., 2015), autism and intellectual disability (Jamain et al., 2003; Laumonnier et al., 2004; Yan et al., 2005; Chen J. et al., 2014). Additionally, the NX/NL complex has been suggested to trigger amyloid deposition in Alzheimer’s disease (Bot et al., 2011; Dinamarca et al., 2011).

All five human and four mammal NL genes have a canonical splicing site A (SS#A; Ichtchenko et al., 1996). NL1 also has a SS#B (Ichtchenko et al., 1996), which acts as a master switch that determines the binding of NLs to NX isoforms (Boucard et al., 2005; Comoletti et al., 2006). NLs are essential for synaptic functions and alterations in their expression can lead to deficits in memory formation and synaptic plasticity (Zhang B. et al., 2015). For example, the overexpression of NL1 shifts synaptic activity toward greater excitation, resulting in impairment in the induction of long-term potentiation (LTP) and deficits in memory acquisition (Dahlhaus et al., 2010). Interestingly, global NL1 depletion also leads to impairments in hippocampal LTP and deficits in spatial learning and memory (Blundell et al., 2010). The acute suppression of NL1 in the amygdala prevents the induction of LTP and results in deficits in the storage of associative fear memory (Kim et al., 2008).

Neuronal activity can induce the cleavage of both NX and NL from their pre- and postsynaptic membranes (Bot et al., 2011; Saura et al., 2011; Peixoto et al., 2012; Suzuki et al., 2012). NLs are cleaved by metalloproteinases (MMPs; Peixoto et al., 2012; Suzuki et al., 2012). Data suggest that NL1 is initially cleaved by MMP9 (Peixoto et al., 2012) and/or a disintegrin and metalloproteinase domain-containing protein 10 (ADAM10; Suzuki et al., 2012), releasing soluble ecto-NL1 and membrane-tethered C-terminal fragments that are further processed by the γ-secretase complex (Peixoto et al., 2012; Suzuki et al., 2012). In addition, accumulation of the soluble extracellular fragment of NL1 leads to a decrease in neurotransmitter release from excitatory synapses (Peixoto et al., 2012; Suzuki et al., 2012). Based on these results, we hypothesized that the cleaved fraction of NL1 activates presynaptic receptors that inhibit transmitter release.

At excitatory synapses metabotropic receptors for glutamate (mGluRs) regulate presynaptic glutamate release and modify postsynaptic excitability to glutamate (reviewed by Niswender and Conn, 2010). mGluRs represent a family of eight known subtypes (mGluR1–8), divided into three groups (I-III; Niswender and Conn, 2010). Group I receptors (mGluR1 and mGluR5) are mainly located postsynaptically where they induce inositol phospholipid hydrolysis. Their activation increases neuronal excitability (Niswender and Conn, 2010). Group II (mGluR2 and mGluR3) and III receptors (mGluR4, -6, -7, and -8) are generally located at presynaptic terminals (Niswender and Conn, 2010). They are coupled to G_i_ proteins that, when activated, prevent the formation of cyclic adenosine monophosphate (cAMP), thereby decreasing the release of glutamate (Niswender and Conn, 2010). Similar to NL1, mutations of mGluR2 are linked to schizophrenia (Li et al., 2015) and autism-like phenotypes in mice (Chen Y. W. et al., 2014). Since both mGluR2 (Niswender and Conn, 2010) and NL1 (Peixoto et al., 2012; Suzuki et al., 2012) regulate the release of neurotransmitters, we decided to examine whether these two proteins regulate transmitter release through functional interactions.

We found that the soluble ectodomain of NL1 activates mGluR2, resulting in a decrease in cAMP formation. In the hippocampus, this pathway inhibits the release of glutamate from mossy fibers that project to CA3 pyramidal neurons.

## Materials and Methods

### Materials

Recombinant rat NL1 (ecto-NL1) was obtained from R&D Systems (Cat. No. 4340-NL-050, Abingdon, UK). Expression vectors that encoded N-terminally HA-tagged mouse NL1-AB, NL1-A, NL1-B and NL1-0 (Addgene plasmid no. 15262, 15227, 15261 and 15260, respectively; Chih et al., 2006) and NL1 without esterase-homology domain (NL1∆ACh; Scheiffele et al., 2000) were gifts from Peter Scheiffele. In the NL1∆ACh deletion mutant, the amino acids 48–630 of murine NL1AB were excised and the HA epitope is followed by the amino acids GVPHLHN. Expression vector that encoded mouse C-terminally mGluR2-green fluorescent protein (GFP) was purchased from Origin (Rockville, MD, USA). Mouse mGluR2 was subcloned into a pcDNA5FRT vector (Life Technologies, Waltham, MA, USA) to create an expression vector that encoded C-terminally strep-tagged mGluR2. The pharmacological inhibitor of mGluR2, RO 64-5229, was purchased from Tocris (Bristol, UK).

### Cell Cultures

Hippocampal neurons were obtained on embryonic day 18–19 from Wistar rat embryos (Charles River Laboratories, Kisselegg, Germany) as described previously (Owczarek et al., 2015). Briefly, the hippocampi were dissected, cleared from blood vessels and meninges, mechanically chopped and trypsinized. The cells were then washed in the presence of DNase and trypsin inhibitor, resuspended, and seeded at a density of 2 × 10^5^ cells/cm^2^ in poly-D-lysine-coated (12 μg/ml) 3 cm Petri dishes (Nunc, Roskilde, Denmark) in Neurobasal medium supplemented with 2% (v/v) B27, 1% (v/v) Glutamax, 100 U/ml penicillin, 100 μg/ml streptomycin, and 2% (v/v) 1 M HEPES (all from Life Technologies). The animals were treated in accordance with the Danish Animal Welfare Act, and the study was approved by the Department of Experimental Medicine at the University of Copenhagen.

Human embryonic kidney 293 (HEK-293) cells were obtained from the European Cell Culture Collection (Salisbury, UK) and maintained in Dulbecco’s modified Eagle’s medium (DMEM) supplemented with 10% (v/v) fetal calf serum, 2 mM Glutamax, 100 U/ml penicillin, and 100 μg/ml streptomycin (all from Life Technologies). All of the cell cultures were kept in a humidified incubator at 37°C that contained 5% CO_2_.

### cAMP Assay

The level of cAMP was measured either in transfected HEK-293 cells or in hippocampal neurons that were grown for 7 days using the commercially available cAMP Parameter Assay Kit (R&D Systems).

HEK-293 cells were plated on 6 cm Petri dishes and transfected using Lipofectamine2000 (Life Technologies) according to the manufacturer’s guidelines with mGluR2-GFP or double-transfected with NL1B-HA and mGluR2-GFP or mock-transfected 48 h prior to the experiment. On the day of the experiment, the cells were washed with phosphate-buffered saline (PBS) and pretreated with 1 mM IBMX (Sigma-Aldrich, St. Louis, MO, USA) for 20 min. Next, the cells were treated with 8 μM forskolin (Sigma-Aldrich) only (double-transfected) or 8 μM forskolin with recombinant ecto-NL1 for 20 min.

Hippocampal neurons were grown for 7 days. To avoid the influence of ionotropic glutamate receptors on cAMP production, the experiments with hippocampal neuronal cultures were performed in the presence of the *N*-methyl-D-aspartate (NMDA) receptor blocker DL-2-amino-5-phosphonopentanoic acid (DL-AP5) and α-amino-3-hydroxy-5-methyl-4-isoxazolepropionic acid (AMPA) receptor blocker NBQX. Additionally, tetrodotoxin (TTX) was used to avoid the indirect effect on cAMP production that could be mediated by cell depolarization (Prezeau et al., 1992). On the day of the experiment, the cells were pretreated with medium that contained 1 mM IBMX (Sigma-Aldrich), 2 μM TTX (Abcam, Cambridge, UK), 20 μM NBQX (Abcam), and 50 μM DL-AP5 (Abcam) for 20 min. Next, the cells were treated with 8 μM forskolin and recombinant ecto-NL1 for 20 min (Kingston et al., 1998). In some experiments, non-competitive mGluR2 inhibitor RO 64-5229 was used. cAMP levels were measured according to the manufacturer’s instructions.

### Coimmunoprecipitation

Rat brains (postnatal day 4–5 [P4–5]) were mechanically homogenized in immunoprecipitation (IP) buffer (PBS with 1% NP40) supplemented with complete protease inhibitors (Roche Diagnostics, Basel, Switzerland). HEK-293 cells were transfected with cDNA expression vectors that encoded the proteins of interest. Forty-eight hours after transfection, the cells were lysed in IP buffer supplemented with complete protease inhibitors (Roche Diagnostics), and cleared lysates were incubated with appropriate antibody overnight. Immunoprecipitates were collected with Pierce protein A/G magnetic beads (Thermo Scientific), washed twice in IP buffer and once with PBS, and analyzed by sodium dodecyl sulfate-polyacrylamide gel electrophoresis and immunoblotting using rabbit anti-mGluR2/3 (1:1000; Millipore, Billerica, MA, USA), mouse anti-NL1 (clone 4C12, 1:1000; Synaptic Systems, Gottingen, Germany), mouse anti-streptag (1:1000; Qiagen), and anti-HA (1:1000; Abcam) antibodies and IRDye (1:20,000; Odyssey, Lincoln, NE, USA) secondary antibodies. The bands were visualized using the Odyssey CLX Infrared Imaging System (Odyssey).

### Slice Preparation

Parasagittal brain slices were obtained from juvenile BL57/6N mice (Taconic) on P8–19. The surgical procedures complied with Danish legislation. This study was carried out in accordance with the recommendations of Department of Experimental Medicine of the University of Copenhagen. The protocol was approved by the Department of Experimental Medicine of the University of Copenhagen. After decapitation, 300 μm slices were cut on a vibratome (MicroM slicer HM 650V) equipped with a cooling unit (CU65), while the tissue was immersed in *N*-methyl-D-glucamine (NMDG) artificial cerebrospinal fluid (aCSF) with the following composition: 125 mM NMDG, 2.5 mM KCl, 26 mM NaHCO_3_, 1.25 mM NaH_2_PO_4_·H_2_O, 3 mM MgCl_2_, and 25 mM glucose bubbled with 5% CO_2_ in 95% O_2_ cooled at 2°C. The slices then rested in oxygenated aCSF of the following composition: 125 mM NaCl, 2.5 mM KCl, 26 mM NaHCO_3_, 1.25 mM NaH_2_PO_4_·H_2_O, 1 mM MgCl_2_, 2 mM CaCl_2_, and 25 mM glucose bubbled with 5% CO_2_ in 95% O_2_ at room temperature for at least 1 h before measurements were performed.

### Patch Clamp Recordings

Measurements were performed at room temperature in a recording chamber that was perfused with oxygenated aCSF. Pyramidal neurons from the CA3 area were visualized by means of a BW51WI microscope (Olympus, Tokyo, Japan) equipped with an oblique illumination condenser. Visually guided patch-clamp recordings of CA3 pyramidal neurons were performed in whole-cell configuration with a Multiclamp 700B amplifier (Molecular Devices, Sunnyvale, CA, USA) in voltage-clamp mode. The pipette solution contained 122 mM K-gluconate, 2.5 mM MgCl_2_, 5.6 mM Mg-gluconate, 5 mM K-HEPES, 5 mM H-HEPES, 5 mM Na_2_ATP, 1 mM EGTA, and 2.5 mM biocytine (pH adjusted to 7.4 with KOH) and the fluorescent dye Alexa 488 or Alexa 568 (10 μM; Sigma-Aldrich). The electrodes had an input resistance of 4–8 MΩ. Fast synaptic transmission that is mediated by NMDA and γ-aminobutyric acid (GABA) receptors was blocked by adding 50 μM DL-AP5 and 10 μM SR 95531 hydrobromide (gabazine). Recordings were sampled at 20 kHz with an analog-to-digital converter** (**DIGIDATA 1440A, Axon Instruments, Union City, CA, USA) and displayed by means of Clampex software.

### Electrical Stimulation

Mossy fibers that originate from the dentate gyrus were stimulated with a bipolar concentric electrode (World Precision Instruments, Sarasota, FL, USA). The stimulus intensity was set between 0.3 mA and 1 mA. The stimulation consisted of a pair of shocks that were applied at 50–300 ms intervals. The effects of NL1 on synaptic transmission were tested by puff-applying ecto-NL1 (4.5 μM) through a glass pipette that was positioned near the apical dendrites of recorded CA3 pyramidal cells. The puff lasted 300 ms and ended 10 ms before the electrical stimulation. For some of the experiments, the mGluR2 receptor antagonist RO 64-5229 (50 μM) was added to the extracellular solution.

### Data Analysis

The amplitude of miniature EPSCs (mEPSCs) was determined by a template-search function (Clampfit, Axon Instruments, Union City, CA, USA).

### Statistics

The normality of samples was tested by means of a D’agostino and Pearson omnibus normality test. Following tests were used: paired and unpaired *t*-test for normally distributed dataset. The non-parametric Kruskal-Wallis test, Friedman’s test, Kolmogorov Smirnov, Mann-Whitney test or Wilcoxon matched-pairs signed rank test were used for non-normally distributed datasets and for small samples (*n* < 10) when Gaussian distribution could not be approximated. The data were analyzed using GraphPad Prism 6 software. Data are represented as mean ± standard deviation of the mean.

## Results

### Recombinant Soluble NL1 Decreased cAMP Levels in Neurons and Heterologous Cells

High neuronal activity results in the intensive shedding of NL1 from the postsynaptic membrane (Peixoto et al., 2012; Suzuki et al., 2012). The accumulation of this soluble extracellular fragment leads to a decrease in synaptic activity (Peixoto et al., 2012), suggesting that NL1 is a part of a negative feedback loop at the synapse. We investigated the mechanism responsible for this process. One possibility is that cleaved NLs interact with presynaptic receptors and thereby regulate neurotransmitter release. We focused on group II mGluRs (mGluR2 and mGluR3) because they regulate neurotransmitter release at excitatory synapses. Upon activation, mGluR2/3 receptors decrease cAMP levels, which produce a reduction of glutamate release and consequently a decrease in postsynaptic activity (Niswender and Conn, 2010). The analysis of mGluR2 and mGluR3 expression pattern reveals that mGluR2 is prominently enriched in all regions of the hippocampus whereas mGluR3 is mainly expressed in CA1 region (Wright et al., 2013).

**Figure 1:**
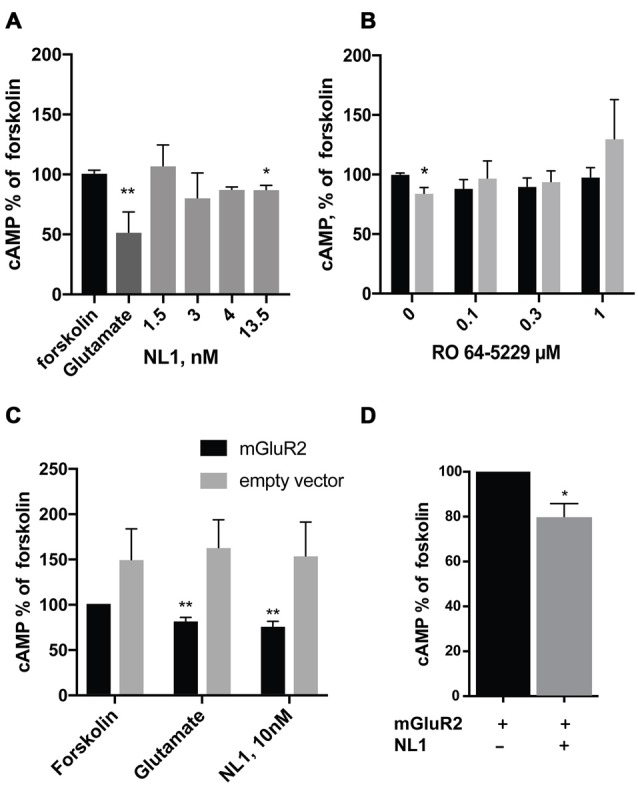
**Soluble neuroligin 1 (NL1) ectodomain reduces cyclic adenosine monophosphate (cAMP) levels via activation of metabotropic glutamate receptor 2 (mGluR2). (A)** Soluble NL1 reduces cAMP levels in neurons. Hippocampal neurons were grown for 7 days and subsequently treated with 8 μM forskolin and different concentrations of recombinant ecto-NL1 for 20 min. Glutamate was used as a positive control. Statistical significance was determined using Kruskal-Wallis test followed by the Dunn’s multiple comparison test *p = 0.0344, **p = 0.01; *n* = 3–7. **(B)** NL1 reduces cAMP levels via activation of mGluR2. Hippocampal neurons were grown for 7 days and subsequently treated for 20 min with 8 μM forskolin only (black bars) or forskolin and recombinant ecto-NL1 (gray bars) in the presence of non-competitive mGluR2 inhibitor, RO 64-5229 in indicated concentrations. Statistical significance was determined using Friedman’s test followed by the Dunn’s multiple comparison test *p = 0.0316, *n* = 4. **(C)** Soluble NL1 activates mGluR2 and decreases cAMP levels. HEK-293 cells were transfected with mGluR2-green fluorescent protein (GFP; black bars) or empty vector (gray bars) and then treated with 8 μM forskolin and recombinant ecto-NL1 for 20 min. Statistical significance was determined using Kruskal-Wallis test followed by the Dunn’s multiple comparison test *p = 0.0049; *n* = 5. **(D)** HEK-293 cells were double-transfected with mGluR2-GFP and NL1B-HA, and then treated with 8 μM forskolin only. Statistical significance was determined using Mann-Whitney test *p = 0.0286; *n* = 4. The results are expressed as a percentage ± SEM relative to the untreated control, which was set at 100%.

We first tested whether the soluble NL1 affects the formation of cAMP in hippocampal primary neuronal cultures. We induced the formation of cAMP with forskolin (Kingston et al., 1998) and tested the effect of extracellular portion of NL1 (ecto-NL1) in the presence of blockers for NMDA and AMPA receptors and voltage-gated sodium channels (see “Materials and Methods” Section). Using competitive enzyme-linked immunosorbent assay (ELISA), we measured cAMP levels in cell lysates. Ecto-NL1 decreased the forskolin-induced formation of cAMP with significant effect observed with 13.5 nM (Figure 1A). To test whether the effect of NL1 was caused by mGluR2 activation, we added the selective non-competitive mGluR2 antagonist RO 64-5229 to the medium. Indeed, in the presence of RO 64-5229, NL1 (13.5 nM) was no longer decreasing the cAMP (Figure 1B). Next, we tested whether ecto-NL1 activates mGluR2 in heterologous cells. We transfected HEK-293 cells with a vector that carried GFP-tagged mGluR2 and quantified changes in the levels of cAMP that were induced by forskolin in the presence of ecto-NL1. Ecto-NL1 decreased cAMP levels in HEK-mGluR2 cells (Figure 1C). Importantly, moc-transfected HEK-293 responded neither to NL1 nor to glutamate (Figure 1C). Additionally, we performed a similar experiment with mGluR3 but did not observe any change induced by ecto-NL1 in cAMP level (data not shown). We therefore focused on mGluR2.

HEK-293 cells express various MMPs (Liu and Wu, 2006; Zhang et al., 2008), including MMP9 and ADAM10, which are involved in NL1 cleavage (Peixoto et al., 2012; Suzuki et al., 2012). Thus, we reasoned that NL1 transfected into HEK-293 might be cleaved and thereby activate mGluR2. Indeed, the cells transfected with both mGluR2 and NL1 exhibited around a 20% decrease in cAMP levels (Figure 1D). These results indicate that NL1 decreases the levels of cAMP in neurons and heterologous cells. The data also suggest that the inhibition of cAMP production by ecto-NL1 involves mGluR2.

### NL1 Interacted Directly with mGluR2/3

**Figure 2:**
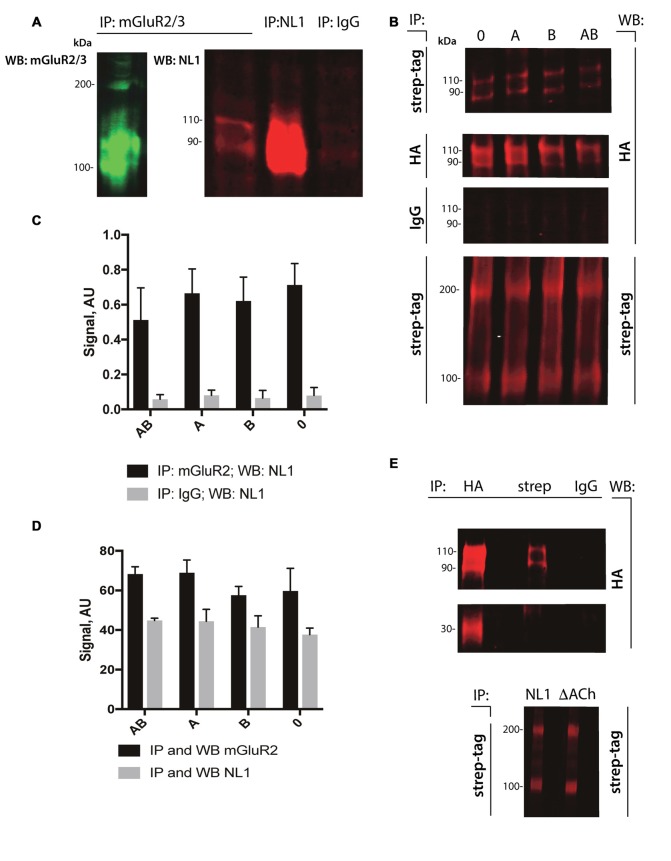
**NL1 interacts with mGluR2. (A)** NL1 was immunoprecipitated from total brain lysates (P4–5) and probed (immunoblotted [WB]) with mGluR2/3 antibodies. Goat normal serum was used as a negative control (IgG). Immunoblots for mGluR2/3 yielded two bands for monomer (100 kDa) and dimer (around 200 kDa). Immunoblots for NL1 also yielded two bands for full-length protein (110 kDa) and a soluble ectodomain (around 90 kDa).** (B)** HEK-293 cells were transfected with HA-tagged NL1 with or without splicing sites A or B and strep-tagged mGluR2 expression vectors. Protein complexes were immunoprecipitated (IP) and probed (WB) with the indicated antibodies. Mouse IgG was used as a negative control. All HA-blots come from the same membrane. Bands at 110 and 90 kDa represent HA-NL1 full length and soluble ectodomain, respectively, and bands at 100 and 200 kDa represent mGluR2 monomer and dimer, respectively. **(C,D)** Quantification of immunoprecipitated NL1 from transfected HEK-293 cells. Bars represent quantified immunoblot signals (*n* = 3). All HA-blots come from the same membrane. **(E)** HEK-293 cells were transfected with HA-tagged NL1 without splicing sites A or B or HA-tagged NL1 without AChE homology domain (∆ACh) and strep-tagged mGluR2 expression vectors. **(Upper panel)** Protein complexes containing mGluR2 and HA-NL1 or mGluR2 and HA-∆ACh were immunoprecipitated (IP) with the indicated antibodies (HA, strep or IgG) and probed (WB) with anti-HA antibodies. Bands at 110 and 90 kDa represent HA-NL1 full length and soluble ectodomain, respectively, band at 30 kDa represent HA-∆ACh. Mouse IgG was used as a negative control. HA-blots come from the same membrane. **(Lower panel)** To confirm the expression of mGluR2 in HEK cells, protein complexes containing mGluR2 and HA-NL1 (NL1) or mGluR2 and HA-∆AChE (∆ACh) were immunoprecipitated (IP) and probed (WB) with anti-streptag antibodies. Bands at 100 and 200 kDa represent mGluR2 monomer and dimer, respectively.

The cleavage of NL1 decreases the release of glutamate (Peixoto et al., 2012). We reasoned that the extracellular portion of NL1 might appear in close proximity to the presynaptic membrane, thus allowing a direct interaction with mGluRs. To test this hypothesis, we performed pull-down experiments from P4–5 rat brain extracts. As shown in Figure 2A, we observed enrichment of NL1 in the brain extracts incubated with antibody against mGluR2/3 compared to IgG control, indicating the existence of mGluR2/3-NL1 complexes in the brain. Importantly, immunoreactivity from NL1 clearly showed two bands with molecular weights around 110 and 90 kDa, respectively, corresponding to the full-length and extracellular domain of NL1 (Peixoto et al., 2012; Suzuki et al., 2012). Similar to the results that were obtained from brain tissue, we observed complexes of HA-tagged NL1 with strep-tagged mGluR2 in transfected HEK-293 cells (Figure 2B). We tested whether the presence of SS#A and/or SS#B in the NL1 sequence affect its interaction with mGluR2. SS#B is unique to NL1 and works as a master switch that determines binding to various NX isoforms (Boucard et al., 2005; Comoletti et al., 2006). The HEK-293 cells were transfected with vectors carrying NL1 with either SS#A, SS#B, both splice sites or none of the splice sites. As seen in Figures 2B–D, the interaction between NL1 and mGluR2 occurs independently from the splice sites SS#A and SS#B. To corroborate the hypothesis that NL1 binds mGluR2 via its extracellular domain, we transfected HEK293 cells with a mutated version of NL1 where amino acids encoding AChE homology domain were excised. NL1 (bands at 110 and 90 kDa), but not NL1 mutant where the AChE homology domain was removed (band at 30 kDa), immunoprecipitated with mGluR2 (IP: anti-streptag antibodies; Figure 2E). This observation confirms that the NL1 ectodomain interacts with mGluR2.

### NL1 Induced Presynaptic Inhibition of Mossy Fibers Contacting Hippocampal CA3 Neurons

**Figure 3:**
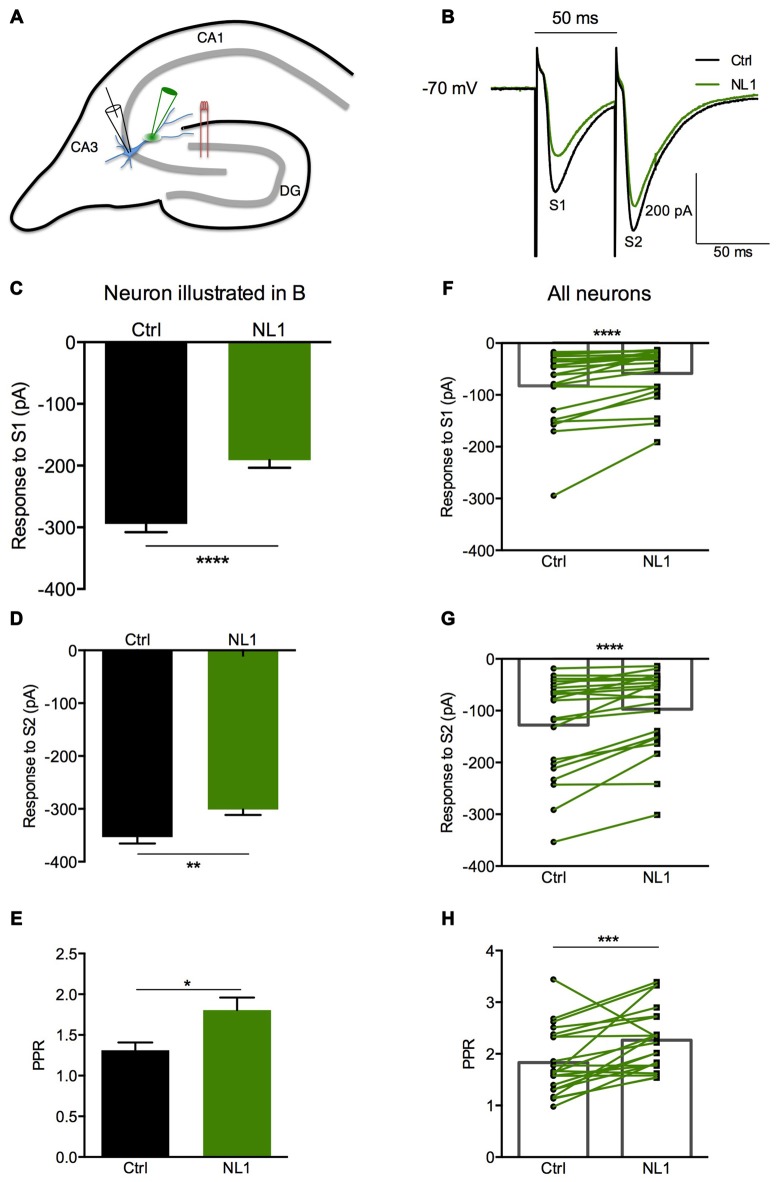
**The NL1 ectodomain produces presynaptic inhibition in CA3 neurons.** **(A)** Schematic diagram of the preparation. Hippocampal CA3 neurons were recorded in voltage-clamp mode (Vh = −70 mV) in whole-cell configuration. The synaptic stimulation of mossy fibers was produced by a bipolar electrode (red), patch electrode (black), and focal puff application of ecto-NL1 (green). *N*-methyl-D-aspartate (NMDA) receptors were blocked by AP5 (50 μM). GABA receptors were blocked by SR 95531 hydrobromide (gabazine; 10 μM). **(B–E)** Response of a CA3 neuron to two stimulations (S1 and S2) under control conditions (black) and after puffing ecto-NL1 near the apical dendrite of the recorded neuron (green). **(C)** The amplitude of the first EPSC decreased from −294.6 ± 13.20 to −191.1 ± 12.46 pA (****p < 0.0001, unpaired *t*-test; *n* = 30 sweeps). **(D)** The amplitude of the second EPSC also decreased but to a lesser extent (from −353.4 ± 12.18 to −301.1 ± 10.47 pA; **p = 0.0019, unpaired *t*-test; *n* = 30 sweeps). **(E)** The PPR, which was calculated as the amplitude of the second EPSC divided by the first, significantly increased (*p = 0.0165, Kolmogorov Smirnov test; *n* = 30). **(F–H)** Results for all cells tested (*n* = 21), with significant decreases for the first (****p < 0.0001) and second (****p < 0.0001) EPSCs (Wilcoxon matched-pairs signed-rank test). **(H)** Significant increase in the PPR for all cells together (***p = 0.0017, paired *t*-test; *n* = 21). In **(F–H)** bars show the mean values and lines correspond to individual pairs of values.

**Figure 4:**
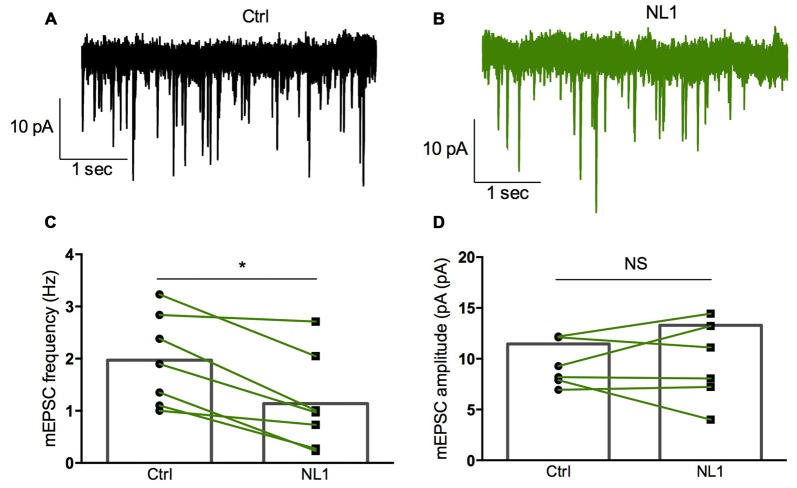
**Miniature EPSCs (mEPSCs) are inhibited by ecto-NL1. (A)** Example of mEPSC recorded in a pyramidal neuron from CA3 region. Five superimposed recordings. **(B)** Recording from the same neuron after a single puff of ecto-NL1 near the apical dendrite of the recorded neuron (five superimposed traces). Note the strong decrease in the frequency of mEPSCs. **(C)** Mean frequency of mEPSCs in control conditions and after ecto-NL1 puff. Significant decrease (mean frequency of mEPSCs in control conditions: 1.97 ± 0.3 Hz; after ecto-NL1: 1.14 ± 0.3 Hz; *n* = 7 cells; *p* = 0.015, Wilcoxon matched-pairs signed rank test). **(D)** No change in the amplitude (mean amplitude of mEPSCs in control conditions: 11.46 ± 2.17 pA; after ecto-NL1: 13.29 ± 3.87 Hz; *n* = 7 cells; one of the pairs is not visible on the plot; *p* = 0.47, Wilcoxon matched-pairs signed rank test. Bars show the mean values and lines correspond to individual pairs of values.

Ecto-NL1 was previously shown to decrease synaptic activity in dissociated hippocampal neurons (Peixoto et al., 2012). To confirm these results in a slice preparation from the mouse brain, we stimulated mossy fiber-CA3 synapses known to express mGluR2 at preterminal levels (Yokoi et al., 1996; Figures 3A,B). We recorded pyramidal neurons from the CA3 region by means of the whole-cell patch-clamp technique. In voltage-clamp mode, with the membrane potential held at −70 mV, we evoked pairs of monosynaptic EPSCs (50–300 ms intervals) by stimulating mossy fibers with a bipolar electrode (Figures 3A,B). A single puff of ecto-NL1 near the apical dendrite of the recorded neuron induced a strong inhibition of the first response (significant decrease for response 1 in 13/21 cells, *p* < 0.05, *n* = 30 for each neuron, unpaired *t*-test; significant decrease for the 21 cells taken together; *p* < 0.0001, Wilcoxon matched-pairs signed-rank test performed on the mean amplitude of the EPSC; Figures 3B,C,F). Ecto-NL1 induced a weaker inhibition of the second response (significant decrease for response 2 in 11/21 cells, *p* < 0.05, *n* = 30 for each neuron, unpaired *t*-test; significant decrease for the 21 cells taken together; *p* < 0.0001, Wilcoxon matched-pairs signed-rank test performed on the mean amplitude of the EPSC; Figures 3B,D,G). Consequently, the paired-pulse ratio (PPR; calculated as the relative amplitude of the second and the first EPSCs) increased (*p* = 0.0017, paired *t*-test; *n* = 21). The PPR increase suggests that the probability of neurotransmitter release was decreased by NL1 (Figures 3E–H). We verified these results by quantifying the occurrence of mEPSCs (Figure 4A). We found that the number of mEPSCs decreased strongly after puffing ecto-NL1 (Figures 4B,C; mean frequency of mEPSCs in control conditions: 1.97 ± 0.3 Hz; after ecto-NL1: 1.14 ± 0.3 Hz; *n* = 7 cells; *p* = 0.015, Wilcoxon matched-pairs signed rank test) while the amplitude was unaffected (Figures 4B,D; mean amplitude of mEPSCs in control conditions: 11.46 ± 2.17 pA; after ecto-NL1: 13.29 ± 3.87 Hz; *n* = 7 cells; *p* = 0.47, Wilcoxon matched-pairs signed rank test). Altogether, our data demonstrate that ecto-NL1 induced presynaptic inhibition of the mossy fiber-CA3 synapse.

**Figure 5:**
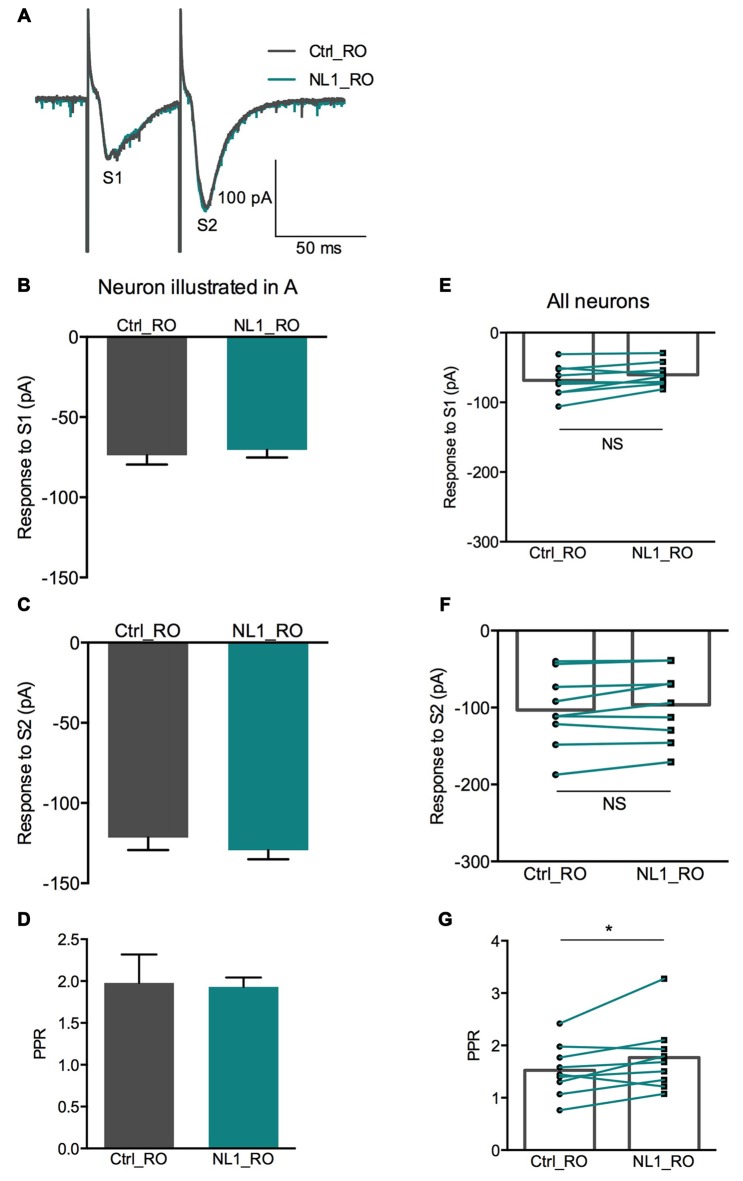
**RO 64-5229 reduces the presynaptic inhibitory effect of NL1. (A)** EPSCs evoked by mossy fiber stimulation in a CA3 neuron in the presence of RO 64-5229 (50 μM) under control conditions (gray) and after one puff of NL1 (teal) near the apical dendrite. **(B–D)** Response of a CA3 neuron to two stimulations (S1 and S2) under control conditions (iron) and after puffing ecto-NL1 near the apical dendrite of the recorded neuron (teal). **(B)** The amplitude of the first EPSC was slightly decreased (from −73.8 ± 5.8 to −70.38 ± 4.9 pA, *p* = 0.65, unpaired *t*-test; *n* = 30 sweeps). **(C)** The amplitude of the second EPSC was slightly increased (from −121.5 ± 7.8 to −129.4 ± 5.7 pA; *p* = 0.40, unpaired *t*-test; *n* = 30 sweeps). **(D)** The PPR was slightly decreased (from 1.98 ± 0.3 to 1.93 ± 0.1; *p* = 0.13, Kolmogorov Smirnov test; *n* = 30). **(E–F)** Results for all cells tested (*n* = 9), no significant decreases for the first (*p =* 0.069) and second (*p* = 0.087) EPSCs (Wilcoxon matched-pairs signed-rank test). **(G)** Significant increase for the PPR for all cells together (from 1.5 ± 0.16 to 1.7 ± 0.22; *p = 0.04, Wilcoxon matched-pairs signed-rank test; *n* = 9). In **(E–G)** bars show the mean values and lines correspond to individual pairs of values.

**Figure 6:**
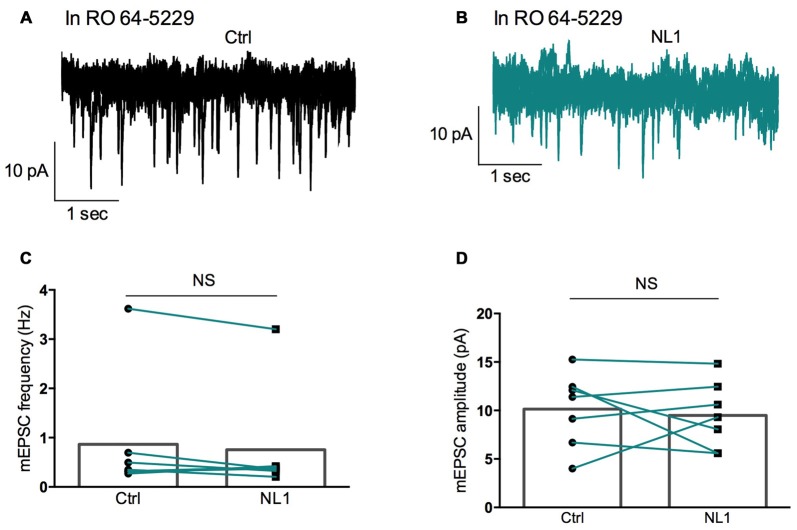
**RO 64-5229 reduces the inhibition of mEPSCs induced by ecto-NL1. (A)** Example of mEPSC recorded in the presence of RO 64-5229 (50 μM) in a pyramidal neuron from CA3 region. Five superimposed recordings. **(B)** Recording from the same neuron after a single puff of ecto-NL1 near the apical dendrite of the recorded neuron (five superimposed traces). **(C)** Mean frequency of mEPSCs in control conditions and after ecto-NL1 puff. No significant decrease (mean frequency of mEPSCs in control conditions: 0.87 ± 0.5 Hz; after ecto-NL1: 0.77 ± 0.41 Hz; *n* = 7 cells; *p* = 0.30, Wilcoxon matched-pairs signed-rank test). **(D)** Mean amplitude of mEPSCs in control conditions and after ecto-NL1 puff. No significant change (mean amplitude of mEPSCs in control conditions: 10.15 ± 1.44 pA; after ecto-NL1: 9.49 ± 1.30 Hz; *n* = 7 cells; *p* = 0.81, Wilcoxon matched-pairs signed rank test). Bars show the mean values and lines correspond to individual pairs of values.

We then tested whether this inhibitory effect of NL1 was caused by the activation of mGluR2 by adding the selective mGluR2 antagonist RO 64-5229 (50 μM) to the extracellular medium. Under this condition, the inhibitory effect of ecto-NL1 on evoked synaptic transmission was strongly reduced (Figures 5A–D; no significant decrease for response 1 for the cells taken together; *p =* 0.069, paired *t*-test performed on the mean amplitude of the EPSC, Figure 5E; no significant decrease for response 2, *p* = 0.087, paired *t*-test, Figure 5F; *n* = 9). In spite of the lack of significant decrease of synaptic transmission, the PPR was increased (from 1.5 ± 0.16 to 1.7 ± 0.22 (i.e., 13%); significant increase, *p* = 0.04, Wilcoxon matched-pairs signed-rank test) but to a lesser extent than in control conditions (from 1.8 ± 0.14 to 2.3 ± 0.13 (i.e., 27%)). In the presence of the mGluR2 antagonist, the ecto-NL1 caused a slight reduction of mEPSCs (Figure 6), though not significant (mean frequency of mEPSCs in control conditions: 0.87 ± 0.5 Hz; after ecto-NL1: 0.76 ± 0.41 Hz; *n* = 7 cells; *p* = 0.3, Wilcoxon matched-pairs signed-rank test). The amplitude of mEPSCs remained unchanged (mean amplitude of mEPSCs in control conditions: 10.15 ± 1.44 pA; after ecto-NL1: 9.49 ± 1.30 Hz; *n* = 7 cells; *p* = 0.81, Wilcoxon matched-pairs signed rank test). Altogether these results show that at least part of the presynaptic inhibition induced by ecto-NL1 was caused by the activation of mGluR2.

## Discussion

Our data suggest that the extracellular soluble fragment of NL1 (ecto-NL1) decreases glutamatergic synaptic transmission by binding to and activating presynaptic mGluR2. This activation prevents the formation of cAMP, which in turn suppresses the release of glutamate (Niswender and Conn, 2010), eventually decreasing synaptic strength. Three arguments support this interpretation. First, NL1 directly bound to mGluR2, demonstrated by the coimmunoprecipitation of NL1 with mGluR2 from transfected HEK-293 cells and pull-down of endogenous brain NL1/mGluR2/3-receptor complexes (Figure 2). Second, NL1 activated mGluR2, demonstrated by our experiments that showed that: (i) ecto-NL1 decreased cAMP levels in hippocampal neurons and this effect disappeared in the presence of mGluR2 antagonist; and (ii) ecto-NL1 decreased cAMP levels in HEK-293 that were transfected with mGluR2 but not in mock transfected HEK-293 cells (Figure 1). Third, the mGluR2 antagonist blocked presynaptic inhibition that was induced by ecto-NL1 (Figures 5, 6). Importantly, mGluR2 are expressed at the pre-terminal zone of mossy fibers that project to NL1-expressing CA3 pyramidal cells (i.e., the cells on which the present experiments were performed; Yokoi et al., 1996; Song et al., 1999). Our data support the idea that NL1 is part of a negative feedback loop that is capable of modifying presynaptic function when postsynaptic activity increases.

Our results show that in the presence of the mGluR2 antagonist, the inhibitory effect of ecto-NL1 was strongly attenuated. However, because blocking mGluR2 receptors did not abolish all the effects of ecto-NL1, other receptors might also be involved in regulation of glutamatergic synaptic transmission by NL1. For example, it is plausible that mGluR3 also interacts with NL1. All mGluRs have high sequence homology (Willard and Koochekpour, 2013). Therefore, the biological effects of soluble NL1 in the brain are likely mediated by various mGluRs, the relative importance of which is defined by their expression patterns. Even though mGluR3 is only moderately expressed in the hippocampus and we did not observe changes in cAMP level in HEK293 cells transfected with mGluR3, we cannot exclude the possibility that mGluR3 might also be involved in the interaction with NL1 in hippocampus. Therefore, mGluR3 might be another candidate for interactions with NL1. Detailed characterizations of these interactions are an interesting line of investigation for future studies.

What is the functional role of NL1-mGluR2 interaction? The activation of mGluR2 reduces the behavioral and electroencephalographic correlates of status epilepticus (Caulder et al., 2014), suggesting a neuroprotective role for mGluR2. In the hippocampus of adult mice, the cleavage of NL1 increases in response to intensive synaptic activity, such as during status epilepticus (Peixoto et al., 2012). Our data show that ecto-NL1 activates mGluR2 in the absence of glutamate (Figures 1C,D) and that NL1 may maintain the activation of mGluR2 after glutamate is taken up. We suggest that the inhibition of synaptic release triggered by the interaction between soluble NL1 with mGluR2 prevents part of the neurotoxic effects that are induced by the massive release of excitatory neurotransmitters (Nairismägi et al., 2004).

## Author Contributions

MDG, EMMC, ABK, OD, AVP, JJ and SO designed and performed experiments; VB, J-FP and SO designed the study; MDG, J-FP and SO wrote the article.

## Funding

The authors would like to thank the Lundbeck Foundation (R171-2014-532 to SO), Dagmar Marshall Foundation, Danish Research Councils, Owensenske Foundation, Simon Fougner Hartmanns Familiefond, Agnes and Poul Friis Foundation, Aase and Ejner Danielsens Foundation, Brødrene Hartmanns Foundation, and Carlsberg Foundation for financial support. The funding sources had no involvement in the planning or preparation of this article.

## Conflict of Interest Statement

The authors declare that the research was conducted in the absence of any commercial or financial relationships that could be construed as a potential conflict of interest.
